# Advances in Vascular Imaging: A Comparative Analysis of Doppler Ultrasound and Multidetector CT for Lower Limb Peripheral Arterial Disease Diagnosis

**DOI:** 10.7759/cureus.62673

**Published:** 2024-06-19

**Authors:** Anbalagan Malaichamy, Vinoth Pandian, Thulasi Arumugam, Chakradhar Ravipati

**Affiliations:** 1 Department of Radiology, Saveetha Medical College and Hospital, Saveetha Institute of Medical and Technical Sciences, Saveetha University, Chennai, IND; 2 Department of Pediatrics, Saveetha Medical College and Hospital, Saveetha Institute of Medical and Technical Sciences, Saveetha University, Chennai, IND

**Keywords:** peripheral artery disease diagnosis, lower limb angiography, peripheral arterial disease, mdct angiography, duplex ultrasound

## Abstract

Background

This study explores the comparison between Doppler ultrasound and multidetector CT angiography (MDCTA) in diagnosing peripheral arterial disease (PAD), emphasizing the urgent need for precise and minimally invasive methodologies in vascular medicine. PAD, stemming from atherosclerosis, manifests as reduced blood flow and symptoms, such as claudication, requiring timely and accurate diagnosis for optimal treatment outcomes. Doppler ultrasound emerges as an option, offering a non-invasive and cost-effective approach. Conversely, MDCTA provides intricate images, albeit with associated risks, such as radiation exposure and potential complications from contrast agents. This research rigorously evaluates the efficacy, safety, and cost-efficiency of these modalities, aiming to provide clinicians with valuable insights for informed decision-making, ultimately enhancing standards of patient care.

Methodology

In this prospective study conducted at Saveetha Medical College, Chennai, 34 patients diagnosed with PAD were enrolled to compare the efficacy of duplex ultrasound and MDCTA in identifying arterial lesions. Statistical analysis comprised kappa statistics and contingency tables to evaluate the concordance between the modalities, with sensitivity, specificity, positive predictive value (PPV), and negative predictive value (NPV) being calculated. Exclusions were made for patients with contraindications to MDCTA, those under 18 years of age, severe renal impairment, and allergies to contrast agents. This research examined the diagnostic accuracy of both imaging techniques, aiming to provide valuable insights into their effectiveness in identifying arterial lesions associated with PAD.

Statistical analysis

This investigation studied the efficacy of Doppler ultrasound and MDCTA in diagnosing PAD, with a particular focus on comparing the accuracy of Doppler ultrasonography (DUS) against MDCTA using sensitivity, specificity, and Cohen's kappa coefficient. Through segmental analysis, valuable insights were garnered into the diagnostic precision of DUS across various arterial segments. The results underscored the significance of DUS as a safe, cost-effective, and non-invasive alternative that complements the utility of MDCTA. This comprehensive assessment sheds light on the comparative strengths of both modalities, offering invaluable guidance for clinicians in selecting optimal diagnostic approaches for PAD assessment. Statistical analysis was conducted using the Statistical Package for the Social Sciences (IBM SPSS Statistics for Windows, IBM Corp., Version 24.0, Armonk, NY).

Results

The sensitivity of ultrasonography (USG) arterial Doppler in evaluating the supra-inguinal, femoropopliteal segments, and infrapopliteal segments was 87.5%, 100%, and 75.32%, respectively. The specificity in evaluating supra-inguinal, femoropopliteal segments, and infrapopliteal segments was 100%, 96.01%, and 83.06%, respectively. The agreement between the two modalities (USG arterial Doppler and CT angiography) obtained by Cohen's kappa analysis with respect to the aortoiliac region and femoropopliteal region was very good (0.91). For infrapopliteal vessels, it was only moderate (0.76).

Conclusion

Duplex ultrasound emerges as an indispensable tool in the investigation of PAD, offering safety, affordability, and non-invasiveness alongside high diagnostic accuracy and substantial concordance with MDCTA.

## Introduction

Lower limb arterial disease significantly impacts the health of middle-aged and elderly populations, presenting with symptoms such as intermittent claudication, ischemic rest pain, ulceration, and gangrene. This condition commonly arises from atherosclerotic narrowing or occlusion in the leg arteries. While intra-arterial contrast angiography has traditionally served as the gold standard for diagnosing such diseases, its drawbacks, including risks associated with arterial puncture, exposure to ionizing radiation, and the potential nephrotoxic effects of iodinated contrast agents, are recognized. Alternative imaging modalities have emerged, including magnetic resonance angiography (MRA), CT angiography (CTA), and Doppler ultrasonography (DUS), each offering its own set of advantages and limitations. Concerns over ionizing radiation and contrast agent use are shared between CTA and contrast-enhanced MRA. However, newer non-ionic contrast agents have been developed to mitigate these effects, offering a safer alternative for patients undergoing these imaging procedures. Nonetheless, DUS remains distinct for its lack of associated risks, making it a preferable option, particularly for patients at risk of potential adverse effects [1].

Lower limb arterial disease significantly impacts the health of middle-aged and elderly populations, presenting with symptoms such as intermittent claudication, ischemic rest pain, ulceration, and gangrene. This condition commonly arises from atherosclerotic narrowing or occlusion of the leg arteries. While intra-arterial contrast angiography has traditionally served as the gold standard for diagnosing such diseases, its drawbacks, including risks associated with arterial puncture, exposure to ionizing radiation, and the potential nephrotoxic effects of iodinated contrast agents, are recognized. Alternative imaging modalities have emerged, including MRA, CTA, and DUS, each offering its own set of advantages and limitations. Concerns over ionizing radiation and contrast agent use are shared between CTA and contrast-enhanced MRA. However, newer non-ionic contrast agents have been developed to mitigate these effects, offering a safer alternative for patients undergoing these imaging procedures. Nonetheless, duplex ultrasonography remains distinct for its lack of associated risks, making it an option, particularly for individuals with concerns about potential adverse effects.

The evolution of DUS technology, encompassing enhancements in transducer technology, image resolution, signal strength, spectral analysis capabilities, and post-processing, has significantly enhanced its utility in the non-invasive assessment of peripheral arterial disease (PAD). These advancements have augmented its ability to visualize accurately and grade vascular abnormalities, providing a safer alternative to conventional methods. Nonetheless, while studies have demonstrated the efficacy of contrast-enhanced multidetector CT angiography (MDCTA) as a viable non-invasive alternative to conventional digital subtraction angiography (DSA) for mapping the vascular tree, comparative analyses between DUS and MDCTA, particularly in the context of PAD, remain limited [2].

Limited comparative literature underscores the motivation behind the current study, which aims to evaluate the accuracy of DUS relative to MDCTA in detecting and quantifying obstructive arterial lesions in the lower limbs. Specifically, the study endeavors to compare the two modalities in terms of their capacity to identify plaque morphology and ascertain the percentage of arterial occlusion. Such a comparison holds significance for advancing our comprehension of non-invasive diagnostic options and potentially informing clinical decision-making in the management of lower limb arterial disease.

## Materials and methods

Materials

This prospective analytical comparative study was conducted over an 18-month period from September 2020 to March 2022 at the Department of Radiology, Saveetha Medical College, Chennai. Thirty-four patients exhibiting clinical signs of PAD were enrolled based on specific inclusion criteria. Exclusions were made for individuals with contraindications to MDCTA, those under 18 years of age, severe renal impairment, and allergies to contrast media. Patients with clinically suspected PAD, regardless of etiology and sex, were included. Factors, such as body habitus, body size and shape, comorbidities (including diabetes, atherosclerosis, hypertension), and smoking status, were also considered.

Methods

The study aimed to compare the efficacy of DUS and MDCTA in diagnosing and quantifying obstructive arterial lesions in the lower extremities. DUS was employed to assess vessel patency and lesion detection, while MDCTA provided comprehensive evaluations of arterial stenosis, occlusion, calcification, plaque morphology, and collateral presence. Procedures adhered to standardized protocols, with DUS studies conducted by a consistent technician to minimize inter-observer variability.

Statistical analysis

Descriptive and inferential statistical analyses were conducted to compare the diagnostic effectiveness of DUS and MDCTA. Descriptive statistics summarized the data, while inferential tests determined significant differences between modalities. A significance level of p < 0.05 was applied. Statistical analysis was conducted using the Statistical Package for the Social Sciences (IBM SPSS Statistics for Windows, IBM Corp., Version 24.0, Armonk, NY), ensuring a robust evaluation of diagnostic capabilities and aiding in the interpretation of findings.

## Results

**Figure 1:**
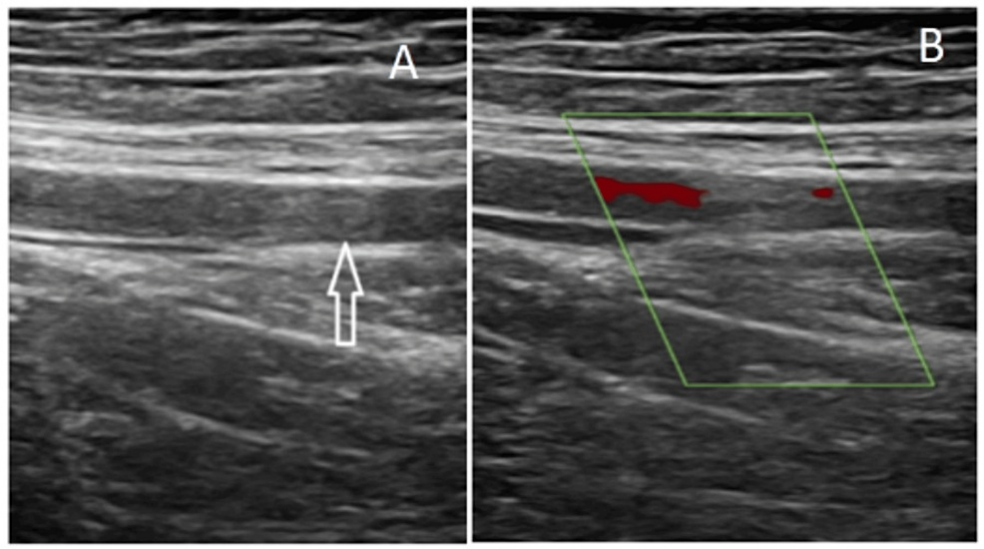
(A) Ultrasonography (USG) B-mode showing iso-echoic plaque in the distal superficial femoral artery (SFA). (B) Color Doppler showing reduced or absent color flow at the thrombosed segment.

**Figure 2:**
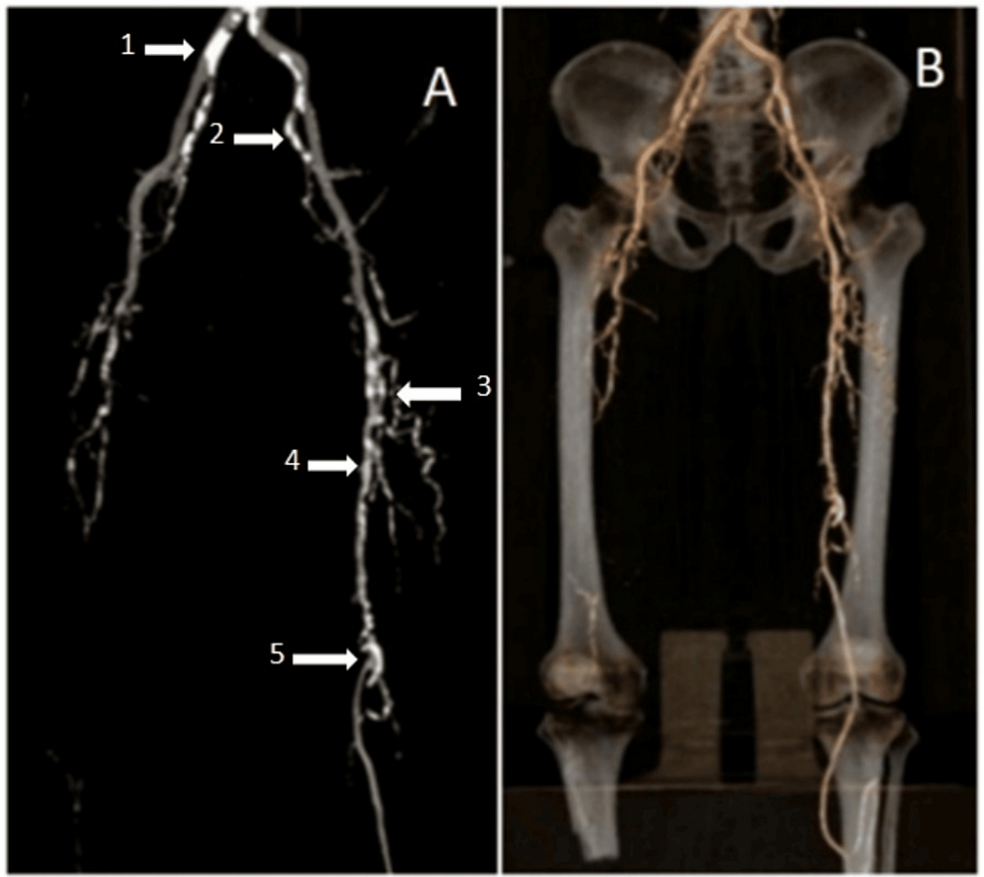
(A) Maximum intensity projection (MIP) image of CT angiography; (B) volume-rendered CT angiography - showing diffuse atherosclerotic vessel wall calcification involving right (1) and left common iliac arteries, right and left (2) internal iliac arteries, left profunda femoris artery (3), left superficial femoral artery (4), and popliteal artery (5).

To diagnose lower limb PAD, DUS and multidetector CT were employed throughout the study. Figure 1 and Figure 2 are example images of a typical DUS scan and CTA, respectively, used to assess blood flow and vessel patency in the lower limbs. This imaging technique allowed for the visualization of arterial flow patterns, which is crucial for identifying areas of stenosis or occlusion.

Figure 1 shows a case of a road traffic accident (RTA) followed by an acute thrombus of the right superficial femoral artery (SFA).

Figure 2 shows maximum intensity projection (MIP) and volume-rendered images.

**Figure 3:**
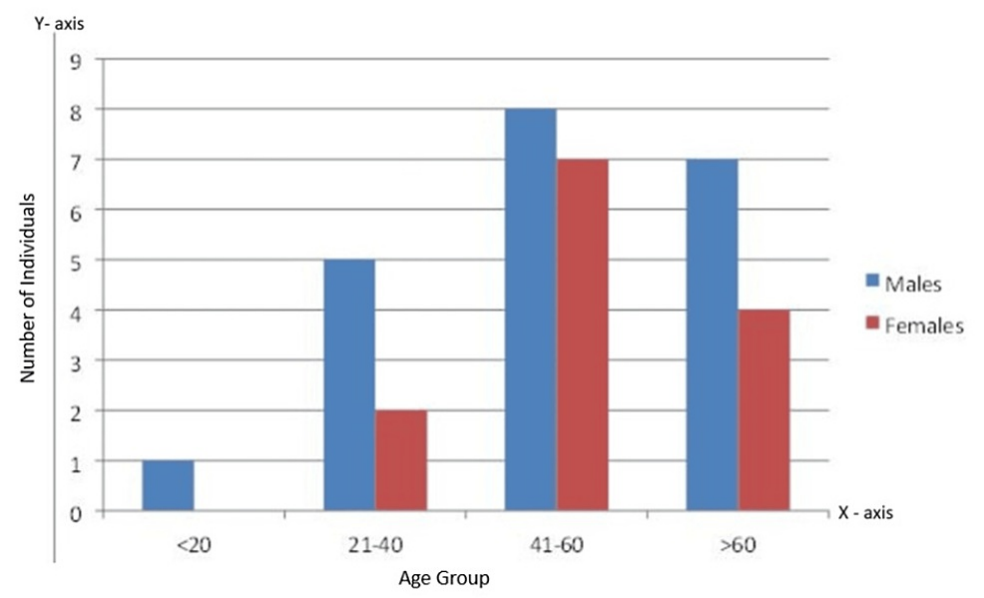
Gender distribution over age group.

This study conducted a segmental analysis of various vascular structures, including the external iliac artery, common femoral artery/deep femoral artery, popliteal artery, anterior tibial artery/dorsalis pedis artery, posterior tibial artery, and peroneal artery. This analysis aimed to assess the presence of hemodynamically significant stenosis or occlusion, explore plaque morphology, and examine collateral circulation. The cohort comprised 34 patients, consisting of 21 men and 13 women. The age distribution revealed one patient under 20 years, seven patients aged between 21 and 40 years, 15 patients aged 41 to 60 years, and 11 patients over 60 years, thus providing a diverse range of age groups for comprehensive vascular evaluation, as depicted in Figure 3.

**Figure 4:**
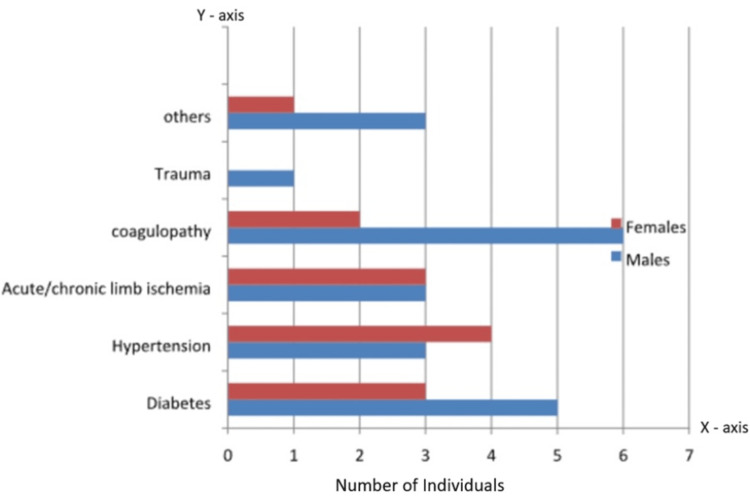
Etiology of peripheral arterial disease (PAD) in the study population.

As depicted in Figure 4, the etiology of PAD in the study group encompasses diabetes, hypertension, limb ischemia, coagulopathy, trauma, and miscellaneous factors. Four patients underwent below-knee amputation. While 68 limbs and 544 individual arterial segments underwent evaluation using each modality, only 524 segments were available for comparison, taking into account the amputated patients. Among the cohort, six individuals exhibited diffuse atherosclerosis, one had thromboangiitis obliterans (TAO), and one presented with hypoplasia of both anterior and posterior tibial arteries. Moreover, eight patients displayed hypercoagulable states attributable to post-trauma, pulmonary thromboembolism, or post-surgery conditions, while an additional eight were diabetic and receiving medication. One patient suffered acute thrombosis due to trauma, and the remaining cases were categorized as miscellaneous. In the miscellaneous category, various factors, such as vasculitis, arteritis, embolism from cardiac sources, or rare genetic vascular disorders, could be implicated. These cases often represent complex or less common presentations of PAD, requiring thorough evaluation and management strategies tailored to individual patient needs. Intermittent claudication was reported by seven patients, rest pain by five, and trophic changes, ulcers, and gangrene were observed in 10 individuals.

During an evaluation involving 68 segments of the external iliac artery, challenges arose when six segments were obscured by bowel gas, narrowing the focus to 62 segments. Within this subset, DUS encountered difficulties in accurately detecting hemodynamically significant stenosis in cases also affected in the common iliac artery, primarily due to CTA's overestimation influenced by atherosclerotic calcifications. As a result, DUS achieved a sensitivity of 87.5% and specificity of 100%, with a perfect positive predictive value (PPV) and a negative predictive value (NPV) of 98.18%. The kappa value of 0.81 signifies substantial agreement. The assessment extended to the common femoral artery, where DUS identified all nine cases of significant stenosis, achieving flawless sensitivity and specificity of 100%, complemented by impeccable PPV and NPV and a kappa value of 0.91, indicating almost perfect agreement. Similar excellence was observed in the evaluation of the proximal and middle segments of the SFA, where Doppler US detected all significant stenoses, maintaining 100% across sensitivity, specificity, PPV, and NPV, with a kappa value of 0.92.

Difficulties arose in assessing the distal SFA, where out of 68 segments, six remained unidentifiable. Despite accurately detecting all significant stenoses in the visible segments, DUS encountered challenges of overestimation in two instances of hemodynamically insignificant stenosis. This was attributed to long-segment disease leading to misinterpreted monophasic spectral patterns. As a result, there was a sensitivity of 100% but a reduced specificity of 92.5%, with a PPV of 94.6%, NPV of 100%, and a kappa value of 0.81. The proximal part of the deep femoral artery, limited by ultrasonography’s (USG's) visualization constraints, exhibited a specificity of 89.83% alongside a sensitivity of 100%, affected by false positives due to calcification-induced overestimation. Evaluation of the popliteal artery through Doppler identified significant stenosis with high accuracy despite minor setbacks from calcification overestimations. Further examination of the tibial arteries revealed complex findings. The anterior tibial artery demonstrated a sensitivity of 75% and a specificity of 86.79%. In contrast, the posterior tibial and deep peroneal arteries encountered similar challenges in achieving congruence between USG and CT findings, as reflected in their respective diagnostic metrics. Despite disparities between Doppler and CTA, the dorsalis pedis artery attained a sensitivity of 74.07% and a specificity of 86.84%. These findings underscore the complexity and necessity of nuanced interpretation in vascular diagnostics, emphasizing Doppler ultrasound’s valuable role in complementing CTA for a comprehensive vascular assessment (Table 1).

**Table 1:** Statistics of the various arteries The lower limb arterial system's sensitivity and specificity with a confidence interval, PPV, and NPV using duplex ultrasound. NPV, negative predictive value; PPV, positive predictive value

Arteries	Sensitivity (%) (95% CI)	Specificity (%) (95% CI)	PPV%	NPV%
Suprainguinal	87.50 (80.37-92.64)	100 (96.63-100)	100	98.46
Femoropopliteal	100 (97.75-100)	96.01 (91.85-98.26)	93.79	100
Infrapopliteal	75.32 (65.94-82.83)	83.06 (75.71-88.65)	65.16	88.88
Overall	91.39 (87.82-93.99)	92.71 (89.77-95.01)	84.47	96.13

Difficulties arose in identifying infrapopliteal vessel reformation in patients with femoropopliteal occlusion due to the presence of numerous collateral vessels. However, DUS revealed that the color flow in infrapopliteal vessels was not opacified by CTA contrast in three patients with significant proximal stenosis. This inconsistency highlighted the limitations of CTA in adequately opacifying vessels beyond occlusions, suggesting that combining USG arterial Doppler with CTA could reduce false-positive occlusion findings (Table 2).

**Table 2:** Agreement between the two modalities - analyzed with Cohen’s kappa analysis USG, ultrasonography

Segment analyzed	Agreement of USG arterial Doppler with CT angiography
External iliac artery	0.81
Common femoral artery	0.91
Superficial femoral artery (proximal)	0.91
Superficial femoral artery (mid)	0.90
Superficial femoral artery (distal)	0.81
Deep femoral artery	0.91
Popliteal artery	0.76
Posterior tibial artery	0.63
Deep peroneal artery	0.61
Anterior tibial artery	0.60
Dorsalis pedis artery	0.61

Therefore, the sensitivity of DUS in assessing suprainguinal, femoropopliteal segments, and infrapopliteal segments was 87.5%, 100%, and 75.32%, respectively. Similarly, the specificity in evaluating suprainguinal, femoropopliteal segments, and infrapopliteal segments was 100%, 96.01%, and 83.06%, respectively, when CTA was considered the gold standard. The agreement between the two modalities (USG arterial Doppler and CTA) was obtained by Cohen’s kappa analysis in the evaluation of various lower limb arteries. Regarding the aorto-iliac region and femoropopliteal region, the agreement was very good (0.91), while that of infrapopliteal vessels was only moderate (0.76) (refer to Table 1 and Table 2).

## Discussion

In cases of extensive calcifications, such as those observed in diffuse atherosclerosis, the diagnostic reliability of CTA is called into question, often exaggerating the severity of occlusion or thrombus. DUS addresses this issue by distinguishing calcific plaques, which may appear to cause more than 50% stenosis. It also reveals color flow in infrapopliteal segments where CT fails due to proximal stenosis. Additionally, DUS improves the assessment of plaque morphology, particularly for soft plaques. USG allows for the determination of occlusion duration, with acute thrombi causing vessel distention and chronic ones narrowing it. Acute thrombi appear hypoechoic or isoechoic with variable echogenicity, some compressibility, and irregular filling defects. Chronic thrombi appear hyperechoic with clear borders and less compressibility and may exhibit acoustic shadowing due to fibrosis or calcifications. In one instance, a patient involved in a road traffic accident suffered a traumatic right SFA injury, revealing complete occlusion on DUS, along with a monophasic spectral pattern distal to the SFA. CTA displayed the occlusion but missed the distended vessel with thrombus due to its inability to reconstruct soft tissue structures. DUS does not use ionizing radiation or iodinated contrast, unlike CTA, which exposes patients to 12-14 mSv of radiation per study. Furthermore, DUS is applicable in emergencies to rule out arterial obstruction without the need for specialized facilities. Doppler's accessibility and cost-effectiveness make it advantageous over other modalities.

In a comparison of the efficacy of MDCTA and DUS for diagnosing mild peripheral arterial occlusive disease (PAOD), a study involving 43 patients with 774 segments who presented with intermittent claudication and leg pain, identified as mild PAOD, underwent DUS followed by MDCTA of the lower limb. The study found that MDCTA detected obstructed or stenotic lesions in 16.8% of arteries, leading to the conclusion that MDCTA could serve as a more accurate screening tool for patients with mild lower extremity PAOD compared to DUS and done by Kayhan et al. [3].

Shirol et al. discovered that MDCTA was statistically significantly better than color DUS in detecting hemodynamically significant stenosis, especially in the femoropopliteal and infrapopliteal segments, as well as in determining the length of blockages, with a p-value of <0.001 indicating high statistical significance [4].

A study by Osama et al. compared the role of multislice CTA against DUS and conventional angiography in assessing aorto-iliac arterial disease. It was found that there was an 82% agreement between DSA and multidetector-row CTA in terms of the degree of stenosis, with a 73% agreement between DSA and color-coded Doppler, highlighting discrepancies mainly due to the enhanced sensitivity of MDCTA in detecting minute amounts of contrast in stenotic segments and the capability of color-coded Doppler to identify weak flow within a stenotic artery [5].

In a study involving 167 patients with advanced PAD, Pinto et al. from the Department of Radiology, University of Pisa, Italy, revealed that arterial Doppler showed a 93.5% diagnostic agreement with MDCT, including accuracies in identifying non-significant stenosis, significant stenosis, and occlusions. The study concluded that USG arterial Doppler is a non-invasive and accurate method for evaluating patients with peripheral ischemic disease [6].

In 2013, Naveen Raj et al. conducted a study focused on 40 patients with clinical signs and symptoms of lower limb PAOD, concluding that MDCT was superior to color Doppler ultrasound in detecting infrapopliteal segment blocks and in the femoropopliteal region in the DFA, with p-values of <0.001 and <0.01, respectively, indicating statistical significance. However, for the SFA, despite better detection rates, the difference was not statistically significant. The study also noted that MDCTA was significantly better than color DUS in evaluating the morphologic features of the runoff arteries at their full length, an important imaging finding. However, the recognition of collateral circulation did not show statistical significance [7].

The results of the present study and those of similar studies evaluating PAD with color Doppler imaging are shown below (Table 3).

**Table 3:** Results of previous Doppler studies in evaluating lower limb arterial system NR, not reported

Study	Patients	Fontaine stage	No. of segments	Doppler positive		Doppler negative		Sensitivity	Specificity
				True+ve	False+ve	False-ve	True-ve		
Aly^9^	90	90/9/1	3108	404	27	34	2643	92.2	99
Bergamini^10^	44	NR	404	94	13	24	273	79.7	95.5
Hatsukami^11^	29	NR	243	73	6	12	152	85.8	96.2
Sensier^12^	76	88/0/12	469	214	26	28	201	88.4	88.5
Legemate^13^	61	80/16/3	918	179	30	33	676	84.4	95.8
Present study	34	2/7/25	807	223	41	21	522	91.39	92.71

A comparative study between DUS and 160-MDCTA in the assessment of chronic lower limb ischemia concluded that both MDCTA and DUS have good predictive value for chronic lower limb ischemia. However, the combination of these two modalities was found to have better diagnostic accuracy [8].

Chidambaram et al. conducted a comprehensive study comparing the efficacy of DUS with CTA in assessing peripheral arteries segmentally. Published in the Journal of Clinical Diagnostic Research, their research aimed to evaluate the effectiveness of DUS, a non-invasive technique, in identifying arterial segments and abnormalities. The study demonstrated a notable level of agreement between DUS and CTA, illustrating DUS's capability to accurately detect various arterial segments, including stenosis, occlusion, and other abnormalities. These findings underscore the significant role of DUS as a non-invasive and valuable tool in evaluating peripheral artery diseases, emphasizing its complementary role alongside CTA in clinical practice [9].

Adiyeke and Karagoz conducted a study comparing the effectiveness of DUS and CTA in predicting amputation level and re-amputation rate. Published in the Northern Clinics of Istanbul journal, their research aimed to assess the capabilities of these imaging modalities in forecasting amputation levels and re-amputation rates in patients. The study revealed that DUS, as a non-invasive and easily accessible technique, showed significant correlations with amputation levels and re-amputation rates. These findings emphasize the potential utility of DUS in clinical decision-making, particularly due to its non-invasive nature and accessibility. It suggests that DUS could play a valuable role in predicting amputation outcomes and improving patient care and management, especially in cases of limb-threatening conditions [10].

In this study, duplex ultrasonography was found to have a high NPV for excluding significant arterial stenosis or occlusion. This capability helps avoid more expensive diagnostic modalities and those involving ionizing radiation in mildly symptomatic patients. Consistent with previous research by Polak et al. (1996) and Geroulakos et al. (1993), our findings highlight DUS's cost-effectiveness and its ability to reduce the need for other costly diagnostic tests. DUS accurately determines the nature and extent of arterial disease, which guides further management - be it surgical, endovascular, or requiring additional imaging, as supported by the work of AbuRahma et al. (1995) and Ricotta et al. (1995). Moreover, our study found that when DUS is used alongside MDCTA, it enhances diagnostic accuracy, especially for equivocal lesions identified by MDCTA, echoing findings by Ota et al. (2004) and Menke et al. (2006). The advantages of DUS over CTA, including the avoidance of ionizing radiation and the provision of real-time hemodynamic assessments, align with the benefits discussed by Iezzi et al. (2009) and Lau et al. (2007). Differences in patient selection, technological advancements, operator expertise, and methodologies across studies may explain any variances in findings. However, our results reinforce the established advantages of DUS in managing arterial diseases.

## Conclusions

This study highlights the efficacy of DUS compared to MDCTA in diagnosing PAD in the lower limbs. DUS is a highly effective non-invasive diagnostic tool, particularly excelling in the femoropopliteal region with 100% sensitivity and 96.01% specificity. It shows substantial agreement with MDCTA in the supra-inguinal and femoropopliteal segments, with Cohen’s kappa values of 0.81 and 0.91, respectively. Although DUS’s concordance is moderate in the infrapopliteal segments, it offers significant advantages in safety and cost-efficiency by avoiding the risks associated with radiation and contrast agents. These attributes make DUS especially valuable for patients who cannot undergo MDCTA. Despite some limitations in regions with complex vessel anatomy or extensive calcification, DUS remains a reliable, safer, and more accessible alternative for vascular assessment and diagnosis in PAD.
